# Learning Hand Kinematics for Parkinson's Disease Assessment Using a Multimodal Sensor Glove

**DOI:** 10.1002/advs.202206982

**Published:** 2023-05-07

**Authors:** Yu Li, Junyi Yin, Shuoyan Liu, Bing Xue, Cyrus Shokoohi, Gang Ge, Menglei Hu, Tenghuan Li, Xue Tao, Zhi Rao, Fanye Meng, Hongfeng Shi, Xiaoqiang Ji, Peyman Servati, Xiao Xiao, Jun Chen

**Affiliations:** ^1^ School of Life Science and Technology Changchun University of Science and Technology Changchun 130022 P. R. China; ^2^ Department of Bioengineering University of California, Los Angeles Los Angeles CA 90095 USA; ^3^ Department of Materials Science and Engineering National University of Singapore Singapore 117583 Singapore; ^4^ Department of Electrical and Computer Engineering National University of Singapore Singapore 117583 Singapore; ^5^ Department of Electrical and Computer Engineering University of British Columbia Vancouver BC V6T1Z4 Canada; ^6^ China–Japan Union Hospital of Jilin University Changchun 130033 P. R. China

**Keywords:** finger flexibility, hand muscle strength, hand stability, Parkinson's disease, smart glove, wearable bioelectronics

## Abstract

Hand dysfunctions in Parkinson's disease include rigidity, muscle weakness, and tremor, which can severely affect the patient's daily life. Herein, a multimodal sensor glove is developed for quantifying the severity of Parkinson's disease symptoms in patients’ hands while assessing the hands’ multifunctionality. Toward signal processing, various algorithms are used to quantify and analyze each signal: Exponentially Weighted Average algorithm and Kalman filter are used to filter out noise, normalization to process bending signals, K‐Means Cluster Analysis to classify muscle strength grades, and Back Propagation Neural Network to identify and classify tremor signals with an accuracy of 95.83%. Given the compelling features, the flexibility, muscle strength, and stability assessed by the glove and the clinical observations are proved to be highly consistent with Kappa values of 0.833, 0.867, and 0.937, respectively. The intraclass correlation coefficients obtained by reliability evaluation experiments for the three assessments are greater than 0.9, indicating that the system is reliable. The glove can be applied to assist in formulating targeted rehabilitation treatments and improve hand recovery efficiency.

## Graphical abstract

This work introduces a glove with multimodal sensors to quantify hand dysfunctions for Parkinson's disease patients. It can capture the hand biomechanical signals and utilizes advanced algorithms for signal processing. The evaluation of the glove's performance demonstrates high reliability, highlighting its potential to aid in devising targeted rehabilitation treatments and enhancing hand recovery efficiency.

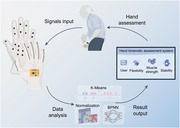

## 1 Introduction

Parkinson's disease (PD) is the leading fastest‐growing neurodegenerative disease around the world.^[^ ^1^ ^]^ The prevalence of PD over the age of 60 is ≈1%,^[^ ^2^ ^]^ which costs healthcare $20 billion annually in the United States.^[^ ^3^ ^]^ The main clinical manifestations of the PD patients’ hands include static tremor, muscle weakness, and rigidity.^[^ ^4^ ^]^ More than 70% of PD patients suffer from static tremor, which is a hallmark of PD.^[^ ^2^ ^]^ Unfortunately, there is no uniform standard test or biomarker to track the disease progression as the symptoms and signs of PD vary with each individual.^[^ ^5^, ^6^, ^7^ ^]^ Clinical scales are the primary method for physicians to examine and evaluate the severity of the neurological signs (such as movement inflexibility, hand muscle rigidity, and tremors) that patients show.^[^ ^8^, ^9^, ^10^ ^]^ Conventional clinical scales include the total active movement (TAM),^[^ ^11^ ^]^ manual muscle testing,^[^ ^12^ ^]^ unified Parkinson's disease rating scale (UPDRS),^[^ ^13^ ^]^ and the movement disorder society criteria.^[^ ^14^ ^]^ However, this method is susceptible to the patient's status during the clinical visit, and to the variability in individual disease characteristics.^[^ ^15^ ^]^ And these scales are imprecise, and lack the ability for the physician to have an objective response during the assessment process, which makes monitoring and assessing PD patients’ hand functions challenging, especially in the milder early stages.^[^ ^16^, ^17^ ^]^ Various efforts have been made in the past to develop suitable flexible data gloves for continuous and objective monitoring and assessment of hand function.^[^ ^18^, ^19^, ^20^, ^21^ ^]^

Wearable sensors hold attractive features such as wearing comfort, excellent compliance, low cost, and light weight.^[^ ^21^, ^22^, ^23^, ^24^, ^25^ ^]^ The combination of wearable sensors and textiles with outstanding air permeability could provide favorable conditions for flexible gloves to track human movement states (joint motion, muscle strength, physiological tremor, and pathological tremor).^[^ ^18^, ^26^, ^27^, ^28^, ^29^, ^30^, ^31^ ^]^ With the support of machine learning feature extraction, subtle features hidden in complex signals can be recognized, and utilized to implement gesture recognition, and hand function assessment.^[^ ^19^, ^25^, ^31^, ^32^, ^33^, ^34^, ^35^, ^36^, ^37^, ^38^, ^39^, ^40^ ^]^ Existing wearable gloves offer high accuracy in monitoring hand movements (Table S1, Supporting Information),^[^ ^19^, ^21^, ^41^, ^42^, ^43^, ^44^, ^45^, ^46^, ^47^, ^48^, ^49^, ^50^, ^51^, ^52^, ^53^, ^54^ ^]^ yet diverse challenges remain to be resolved. On the one hand, since the majority of research only utilizes a single sensor to capture limited indexes to characterize hand function, this situation is farfetched to characterize the diverse motion characteristics of the hand.^[^ ^18^, ^19^, ^20^, ^22^, ^34^, ^44^ ^]^ On the other hand, researchers were more interested in capturing hand movement information and neglected to incorporate scales for clinical assessment.^[^ ^34^, ^38^, ^44^, ^47^, ^48^ ^]^ Combining objective measurements and clinical scales can promote the assessment of hand dysfunction, and guide physicians in the development of treatment plans for hand rehabilitation.^[^ ^43^, ^45^, ^46^ ^]^

Herein, we report a multimodal sensor‐based textile glove for monitoring and assessing PD patients’ hand function. The glove comprehensively obtains information of various hand kinematics through a built‐in flexible sensor system. Specifically, flexible sensors measure the bending angle of the fingers and the force of fingers and palm applied on objects. Combined with hand tremor signals fed back by accelerometers, our glove analyzes the participants' hand functions in an objective and exhaustive manner with comfortable wearable experiences for patients. Furthermore, a large scale of data‐containing hand kinematics was captured from 40 participants using the multimodal sensor glove, which was shown capable of quantitatively assessing finger flexibility, hand muscle strength, and hand stability with the aid of filtering, normalization, cluster analysis, and neural network. Finally, we proved the consistency of the data by comparing the assessment results and clinical evaluation with Kappa values, and we gathered a visualization of the results with human–machine interaction (HCI) equipment. Our remarkable system that combines comfort experience and comprehensive functions, offers a distinctive universal approach to long‐existing challenges in hand function assessment in PD patients. In addition to aiding in formulating rehabilitation treatments, our glove can objectively assess patients’ progress following hand rehabilitation training and direct physicians in making prompt treatment adjustments.

## 2 Results and Discussions

### 2.1 Design of the Multimodal Sensor Glove

The primary components of the system are the sensor system, the primary control module, and the PC terminal. A proper selection of sensors is crucial for achieving comprehensive hand function assessments. The chosen sensor system must be capable of accurately capturing various hand kinematic information while meeting requirements for comfort and flexibility. Embedding too many rigid sensors not only increases the cost of the glove but may also limit patients’ freedom of movement, thus reducing comfort and convenience. Flexible sensors can effectively solve this problem by sensing multiple physical quantities within a small area and better adapting to the shape and movement of the hand. Flexible bending sensors, with a resistance range of 10–110 kΩ and a resistance tolerance of ±30%, were selected to measure finger bending angle information. Flexible thin‐film pressure sensors, with a sensitivity of ≤10 g and an accuracy of <5%, were chosen to measure changes in hand muscle strength. Inertial Measurement Unit (IMU) sensors were chosen to measure the acceleration signal generated by hand tremors due to their high precision and programmable acceleration range characteristics (ranging from ±2 *g*, ±4 *g*, ±8 *g*, to ±16 *g*, with *g* denoting gravity acceleration). In addition, the microcontroller unit (MCU) selected can meet the interface and processing speed requirements of the system.

**Figure 1:**
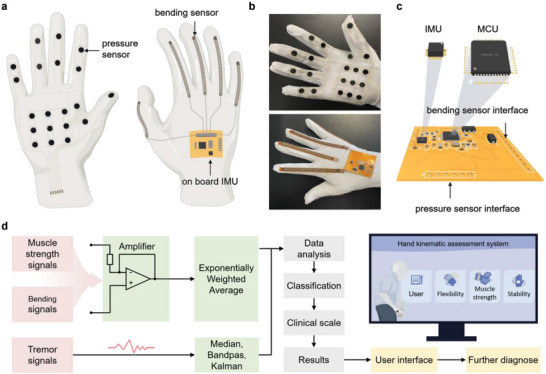
Multisensor‐based hand function assessment multimodal sensor glove. a,b) A depiction of the inner layering of the multimodal sensor glove and the layout of each sensor. Flexible film pressure sensors are located on the palmer side of the glove, with detection points corresponding to the palm of the hand, purlicue, middle phalanges, and distal phalanges. Flexible bending sensors are located on each of the dorsal phalanges. IMU and MCU are integrated onto the dorsal side of the glove. c) The 3D diagram of the main control circuit board, including the physical interfaces connected to MCU, IMU, bending sensors, and thin film pressure sensors. d) Hand function assessment process and HCI. The hand function assessment process includes collecting, processing, and analyzing hand kinematic signals.

The proposed multimodal sensor glove features a two‐layer structure with an outer layer protecting the sensors from the environment and an inner layer offering the sensors positioning and support. The layout of the sensors is based on pathological investigations of the PD patient's hands. To quantify hand muscle strength values, we situated the flexible thin‐film pressure sensors in the palm of the multimodal sensor glove, corresponding to specific human hand positions like palm, purlicue, distal phalanx, and middle phalanx (**Figure** **1**a,b). Five bending sensors are placed on the back of the glove's fingers (Figure 1a,b) to capture the bending variations of fingers. In addition, the IMU on the main control circuit board (Figure 1b; Figure S1, Supporting Information) is fitted to the back of the multimodal sensor glove (Figure 1a,b) to measure the acceleration caused by hand tremors.

Figure 1c depicts the main control circuit board for the multimodal sensor glove system. The main board incorporates the minimum system of the MCU, the bend sensor module circuit, the pressure sensor module circuit, the IMU sensor and its peripheral circuit, and the power supply module (Figures S2–S6, Supporting Information). In addition, physical interfaces are reserved on the main control circuit board for bending and pressure sensors to facilitate data transfer and subsequent data processing. Figure 1d demonstrates the hand function assessment. Quantitative processing and analysis are performed based on the collected hand kinematics data. After appropriate preprocessing (amplification and filtering) steps, each signal is quantitatively analyzed. Bending signals are normalized to simplify the grading mechanism, while hand muscle strength signals are sorted using the cluster analysis method. Then, a Back Propagation Neural Network (BPNN) is applied to identify and classify tremor signals. The hand function assessment results are then determined by comparing the assessment data to the clinical scale, followed by visualization through HCI interface (Figure S7, Supporting Information).

### 2.2 Finger Flexibility Assessments

Hand flexibility is determined by the flexion of phalanges, which are influenced by the mobility of synovial joints within the metacarpophalangeal and proximal/distal interphalangeal joints. Disorders of the nervous system caused by PD can lead to symptoms such as joint rigidity and muscle disuse atrophy, which results in decreased flexibility and range of motion of the fingers due to inflammation and fibrosis of the synovial joints. We collected participants' finger bend signals via the flexible bending sensor module and quantitatively analyzed the differences in hand flexibility.

**Figure 2:**
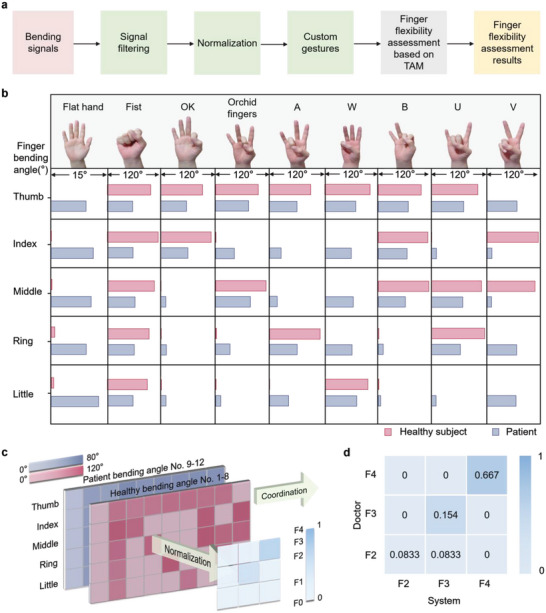
Finger flexibility assessment. a) The process of the flexibility assessment. b) The finger bending angle of two subjects (patient and healthy subject) when completing nine assessment gestures (“Flat hand”, “Fist”, “OK”, “Orchid fingers”, “A”, “W”, “B”, “U”, “V”). c) Schematic diagram of the hand function assessment for 12 subjects. d) Heat map of hand flexibility assessment grades distribution given by the system and the doctor, respectively. The row lists grades given by the system, while the column lists grades given by the doctor.

During the hand flexibility assessment (**Figure** **2**a), each subject wore the multimodal sensor glove and completed nine gestures “Flat hand”,“Fist”,“OK”,“Orchid fingers”,“A”,“W”,“B”,“U”, and “V” in sequence according to the prescribed protocol. Each gesture was performed ten times with a 3‐s interval to reduce measurement errors caused by finger fatigue. The multimodal sensor glove collected the finger‐bending data once each gesture was completed. Figure 2b and Table S2 (Supporting Information) show gesture completion by two subjects (one patient and one healthy subject), indicating a significant difference in the bending angle of fingers between the two subjects. It can be seen that the patient with PD suffers hand dysfunction in both flexion and stretching of the fingers. For the “Flat hand” gesture, the patient's finger curvature is ≈100 times than that of the healthy subject's, indicating that the patient's fingers face certain obstacles in stretching. For the “Fist” gesture, the patient's finger curvature is ≈0.5 times than that of the healthy subject's, indicating that the patient's fingers have dysfunction in flexion. For the “OK” gesture, the bending angles of the patient's thumb and index fingers are ≈0.5 times than that of the healthy subject's, and the bending angles of the patient's middle, ring, and little fingers are about ten times than that of the healthy subject's, indicating that the patient's fingers have dysfunction in flexibility and coordination. The difference in finger flexibility between the two subjects is as apparent when comparing other gestures. Therefore, it can be concluded that the patient suffers from dysfunction in hand flexibility compared to the healthy subject.

Figure 2c illustrates a schematic diagram of the multimodal sensor glove system, with the hand flexibility grades of each subject given in Table S3 (Supporting Information). To compare the consistency of the flexibility assessment results with clinical observations, the clinical scale used by doctors for hand flexibility assessments was also applied to the subjects (Table S3, Supporting Information). The distribution of grading results of 12 subjects given by our system and doctors is analyzed and further illustrated in a heat map (Figure 2d), indicating that the grade F4 is the largest, accounting for 66.7%. About 8.33% of total cases reports differences between the grading results given by our system and doctors. The results indicate that the flexibility grades of subjects 1 to 8 (healthy subjects) are distributed in the grade F4, and the flexibility grades of subjects 9 to 12 (patients) are distributed between F3 and F2. The final Kappa value calculated by the Kappa consistency test is 0.833 (Z‐score of 3.906, *P < 0.05, **P < 0.01), indicating that the flexibility grades given by the system and clinical observations are highly consistent.

### 2.3 Hand Muscle Strength Assessments

Early symptoms of PD such as hand muscle weakness, rigidity, and muscle pain, are often mistaken as common representations of aging. However, with the aggravation of the diseases, the nerve conduction velocity of the patient's upper extremity decreases due to demyelination of the nerves, gradually weakening the sensory function of the median and ulnar nerves. Therefore, the hand muscle strength test is necessary for judging the extent of damage to hand muscles and nerves, which is significant in monitoring the progression of PD. To assess the patient's muscle strength status, we collected muscle signals during hand touching objects via the flexible thin‐film pressure sensor, followed by a series of quantitative analyses to measure the damage level of muscle strength.

**Figure 3:**
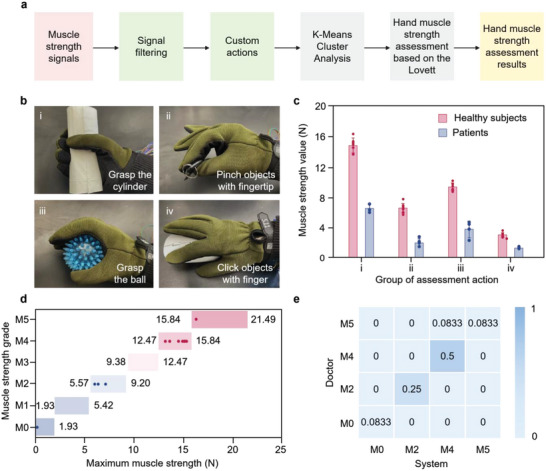
Hand muscle strength assessment. a) The process of the muscle strength assessment. b) Four sets of customized assessment actions: i) grasp the cylinder, ii) pinch objects with fingertip, iii) grasp the ball, and iv) click objects with finger. c) The muscle strength status of 12 subjects completing customized actions. d) Interval partitioning results of cluster analysis. The ordinate represents grades, while the abscissa represents the range of the interval, given the specific values of the interval boundaries. e) Heat map of muscle strength assessment grades distribution given by the system and the doctor, respectively. The row lists grades given by the system, while the column lists grades given by the doctor.

During the muscle strength assessment, each subject wore the multimodal sensor glove and completed four groups of customized actions (**Figure** **3**a,b). We required all subjects to perform each action ten times and ensured that they had a 3‐s rest period to improve the accuracy of the measurement results. Figure 3c shows the corresponding muscle strength values of 12 subjects who completed these actions. Significant differences in hand muscle strength values are shown between patients and healthy subjects. However, the action of “grasp the cylinder” requires the most physical contact between the hand and the sensors, demonstrating the maximum values of measured muscle force. Table S4 (Supporting Information) indicates the specific muscle strength of each subject when completing each action. Subject 9 exhibited weakness when performing the actions of “grasp”, “pinch”, and “click”, showing significantly lower corresponding muscle force values. Subjects 10, 11, and 12 demonstrated different extents of strength dysfunction when completing the actions.

Combined with the interval partitioning results of cluster analysis (Figure 3d) and the Lovett grading standard, the hand muscle strength of the subjects was assessed with the maximum muscle strength values collected. Table S5 (Supporting Information) shows the muscle strength value of each subject. To compare the consistency of the hand muscle strength assessment results with clinical observations, the clinical scale was applied for hand muscle strength assessment by doctors (Table S5, Supporting Information). The distribution and proportion of grading results were analyzed and further illustrated in a heat map (Figure 3e), showing that the number of subjects with the hand muscle strength grade M4 is the largest (50%). About 8.33% of the cases report differences between the grading results. The results show that the hand muscle strength grades of subjects 1 to 8 (healthy subjects) are distributed between M4 and M5, and the hand muscle strength grades of subjects 9 to 12 (patients) are distributed between M0 and M2. The final Kappa value calculated by the Kappa consistency test is 0.867 (Z‐score of 4.637, *P < 0.05, **P < 0.01), indicating highly consistent hand muscle strength grades given by our system and clinical observations.

### 2.4 Hand Stability Assessments

The most prevalent tremor in early PD is a unilateral finger rolling movement, which then evolves into uncontrollable, regular tremors of the ipsilateral or contralateral limbs in a stationary condition. Although hand tremors will not cause direct harm from a physiological standpoint, they create significant disruption in everyday living. As a result, measuring tremor signals is critical in rehabilitating patients' hand functions. Recently, accelerometers have been one of the most common methods for measuring tremor signals, and machine learnings are also frequently applied to identify and classify tremors.^[^ ^55^, ^56^ ^]^ To quantify the tremor degree of the patients' hands, we collected acceleration signals via IMU sensor, followed by extracting characterizations (amplitude, standard deviation, frequency, etc.). The tremor data were then classified by using BPNN^[^ ^57^ ^]^ and tenfold cross‐validation,^[^ ^58^ ^]^ which was used to assess the patients' hand stability.

**Figure 4:**
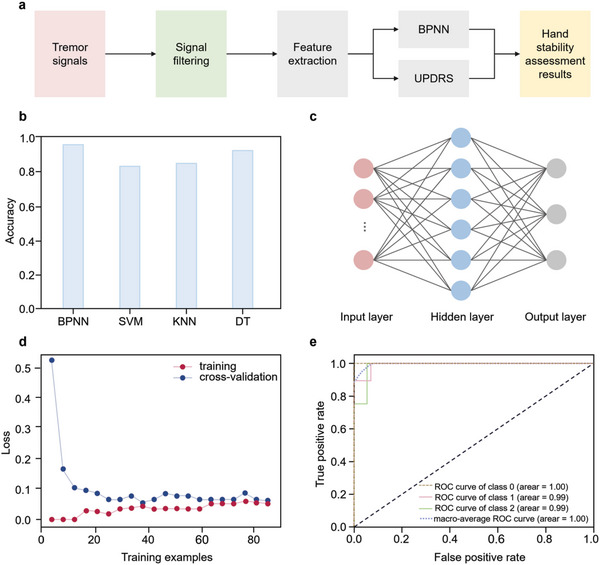
Hand stability assessment. a) The process of the stability assessment. b) Comparison of different methods in terms of accuracy. c) Schematic diagram of the BPNN: nine input layer nodes, six hidden layer nodes, and three output layer nodes. d) Loss function of BPNN training. e) ROC curves for stability assessment based on BPNN. The horizontal coordinate of the ROC curve is not correlated with the vertical coordinate, and the closer the ROC curve is to the (0, 1) point, the better the model is represented. Area under curve (AUC) is the area enclosed by the ROC curve and the *x*‐axis. The value of AUC can be used to measure the goodness of the classifier, and the higher the AUC means the better the classification effect.

**Figure** **4**a shows the stability assessment. In the test, based on the Static Tremor section in UPDRS, each of the 40 subjects was required to wear the multimodal sensor glove and complete a palm‐down motion to remain the hand horizontally for 5 s. The doctors scored the grade of hand tremor of each subject. We labeled the samples as “0”, “1”, and “2” for “No tremor”, “Mild tremor”, and “Severe tremor”, respectively. Furthermore, to avoid the overfitting phenomenon, the original sample of 40 groups was expanded to 120 groups (Table S6, Supporting Information). Specifically, we designed a simulated tremor experiment in which 32 healthy subjects were required to randomly simulate different grades of tremor at frequencies ranging from 3 to 8 Hz according to the tremor motion characteristics. In addition, the acceleration data generated by the hand at different stages were also recorded in eight PD patients.

We performed feature extraction on 120 sets of samples. And the obtained feature values and the labels labeled by the doctors were used to train the machine‐learning model. Then, we partitioned all experimental samples into training and test sets with 8:2, and used tenfold cross‐validation to determine the optimal hyperparameters of BPNN, Support Vector Machine, K Nearest Neighbors, and Decision Tree classifiers (Tables S7–S10, Supporting Information). Compared with other models above, BPNN with the number of hidden nodes of six obtained a higher accuracy of 94.78% (Table S7 and Figure S8, Supporting Information).

Finally, we verified the generalization ability of each classifier on the test sets, and the comparison results showed that the BPNN got the highest accuracy (95.83%), which is shown in Figure 4b and Table S11 (Supporting Information). In addition, Figure 4d shows the loss function of the BPNN training, the final trained loss function value is approaching 0.1. And we evaluated the classification effectiveness of the classifier using receiver operating characteristic (ROC) curves (Figure 4e). The results show that the classifier performs well. The trained confusion matrix is shown in Figure S9 (Supporting Information). We analyzed the values of precision, recall, and F1 score. Experimental results show a good classifier performance in precision and recall in the “No tremor”, “Mild tremor,” and “Severe tremor” categories. Specifically, the precision and recall of the “No tremor” category are both 100%; the precision of the “Mild tremor” category is 100%, and the recall is 90%; the precision of the “Severe tremor” category is 85.71%, the recall is 100%. Considering both recall and precision, we calculated the F1 scores of each category. The F1 scores for the categories “No tremor”, “Mild tremor”, and “Severe tremor” were 100%, 94.74%, and 92.31%, respectively, indicating a good classifier performance across all classification categories. The classifier is very accurate in classifying samples into the correct category. Furthermore, a higher Kappa value is obtained by the Kappa consistency test, which is 0.937 (Z‐score of 6.473, *P < 0.05, **P < 0.01), indicating a strong consistency between the stability assessment and the doctor's assessment.

### 2.5 System Reliability Analysis

We conducted three repeated reliability analysis experiments to comprehensively validate the reliability of the multimodal sensor glove for assessing hand dysfunction. The reliability analysis experiments include finger flexibility assessment, hand muscle strength assessment, and hand stability assessment. 12 subjects (8 healthy subjects and 4 patients) were required to complete three experiments sequentially, each repeating ten times in the same time period. To avoid subject fatigue during experiments, we provided a 1‐min rest period between each experiment, and the rest duration was adjusted according to the subjects' condition. The intraclass correlation coefficient (ICC) model calculated the ICC value and 95% confidence interval of each repeated experiment. The results, presented in Table S12 (Supporting Information), showed that the ICC values for finger flexibility, hand muscle strength, and hand stability assessments were 0.923, 0.91, and 0.946, respectively. Overall, the ICC values of the multimodal sensor glove system were greater than 0.9, indicating its high reliability in evaluating hand dysfunctions. The multimodal sensor glove provides an objective, effective, and comprehensive tool for clinical evaluation and objective quantitative data for subsequent rehabilitation treatments.

## 3 Conclusion

A multisensor‐based multimodal glove for PD patients has been fabricated, with high comfort, low cost, and variable size characteristics. The multimodal sensor glove captures and analyzes the motion signs of rigidity, muscle weakness, and tremor in the patient's hand, which bring a breakthrough in assessing the hand function of PD patients. To examine the subjects' hand flexibility and coordination levels, different gestures were designed for one‐fingered, two‐fingered, and five‐fingered situations. We designed hand grasping, pinching, and clicking actions for specific hand parts like finger pulp, fingertips, palm, and purlicue to illustrate the hand muscle strength condition. The hand tremor grade was examined by analyzing the Static Tremor section in UPDRS. Our results indicated that the multimodal sensor glove recorded the signals on bending angles, muscle strength values, and acceleration generated by tremors when completing designated gestures/actions. Graded results of hand dysfunction assessment were generated after analyzing the above signals, which can be used to assist in assessing the development stages of the disease. Furthermore, the reliability of the system for finger flexibility, hand muscle strength and hand stability assessment was confirmed by repeated reliability evaluation experiments, respectively, indicating that the system has excellent reliability.

The proposed multimodal sensor glove demonstrates the ability to monitor and assess signals on hand bending angles, muscle strength values, and tremor grades in real‐world scenarios by utilizing a multisensor system. The assessment techniques and methods are well aligned with current clinical rehabilitation needs, addressing the noncomprehensive nature of current hand function assessment devices. Various useful signals and graded results on the patient's hand function are provided, assisting doctors in formulating rehabilitation treatment plans for the disease. In addition, the future topic on improving the functionality of the multimodal sensor glove includes developing procedures combining hand function assessment and the rehabilitation process. A detailed study on the relationship between various kinematic parameters of each finger joint during hand movements is required to further facilitate a comprehensive hand function assessment.

## 4 Experimental Section

### Fabrication of Electronics

Flexible printed circuit (FPC) was patterned using a UV laser system (LPKF; Protolaser U4) for mechanical support and electrical interconnections of electronic components. The FPC has dimensions of 43 mm * 38 mm and contains passive components (resistors and capacitors), microcontroller (STM32F103C8T6), DC–DC converter (AMS1117‐3.3 V), operational amplifier (LM358), and IMU (MPU‐9250). The IMU is connected to the FPC through a low‐temperature reflow process with a soldering paste and a heat gun. Flex 4.5″ flexible bending sensors and ZNS‐01 flexible thin‐film pressure sensor are electrically connected by soldering thin copper wires with the FPC reserved interface, respectively.

### Ethics Information and Study Design

This study was approved by the ethics committees of China–Japan Union Hospital of Jilin University (Clinical Research Review No. 20221124002). All participants gave written informed consent for the research to track and analyze information on various hand kinematics. Inclusion criteria of patients: 1) diagnosed with PD, 2) age above 60, and 3) has enough cognitive and verbal ability to understand experiments and follow instructions. Exclusion criteria: 1) behavioral and cognitive impairment and/or low adherence, and 2) diagnosed with other neurological diseases. Eight eligible patients with different severity of hand dysfunctions were recruited in this study. Their symptoms contain but were not limited to rigidity, inflexibility, and tremor, with an average nine‐year illness time. In addition, 32 healthy subjects with no neurological or other related diseases were control groups (Table S13, Supporting Information). Eight healthy subjects and four patients with PD were chosen for hand flexibility and muscle strength assessment tests. Based on the sample size, all 40 subjects took the stability assessment test. Following the test requirement or instruction, each subject was asked to perform corresponding gestures or actions, which the system then collected, processed, and analyzed the gesture/action data to complete the hand function assessment.

### Finger Flexibility Assessment

As bend signals were analog, low‐frequency signals, they must first be processed by analog to digital conversion (ADC) and amplified to acquire target data for the system to analyze. To eliminate the noise mixed in with the target signal, the Exponentially Weighted Average algorithm was applied to filter signals. The algorithm banished the negative effect over responseof electronic devices and greatly neutralized the beating of the data caused by the environmental disturbance (Figure S10, Supporting Information). Furthermore, the min–max standard method was applied to normalize collected bending data, which varied by the individual difference in a range of 0° to 180°. After mapping the bending data to the region of (0, 1), the primary dimension of the signals was eliminated, and the data were dimensionless, which simplified subsequent rating work. Table S14 (Supporting Information) shows the normalized bending angle range with its corresponding grade. A total of five grades (F0 to F4, where F0 indicates the worst flexibility) were refined based on the four grades of the TAM assessment standard (Table S15, Supporting Information). The fingers' ability of flexion and extension was then explored through the designed gestures (“Flat hand”, “Fist”, “OK”, “Orchid fingers”, “A”, “W”, “B”, “U”, “V”). It is worth noting that the maximum bending value obtained through several measurements and computations would be used for the following ranking task. Based on the traditional TAM scale, after comparing the maximum bending values with Table S14 (Supporting Information), the severity of patients' fingers flexibility and corresponding grades were obtained, which were displayed over HCI interface, simultaneously (Figure S7b, Supporting Information).

### Hand Muscle Strength Assessment

During quantitative analysis, the collected muscle signals were processed through ADC, amplification, and filtration. In the filter process, the Exponentially Weighted Average algorithm effectively diminished noise that was caused by factors such as hand tremors or long‐term bending of the sensor (Figure S11, Supporting Information). Additionally, the experiment designed four sets of actions (“Grasp the cylinder”, “Pinch objects with fingertip”, “Grasp the ball”, and “Click objects with finger”) to investigate the pinch strength and grip strength as well as the muscle strength of the finger pulp, fingertips, palm, and purlicue (the muscle between forefinger and thumb). Next, the idea of Cluster Analysis was put forward, which calculates the scalability and efficiency of the operation while keeping the data distance, classifying the grade of muscle strength at a data‐based angle, and obtaining the optimal solution in the local range by using the distance from the measured muscle strength data to the set a classification center point. K‐Means Cluster Analysis was chosen for dividing the muscle strength grades.^[^ ^59^ ^]^ The *k* points were selected as the initial centers (*μ*_1_,*μ*_2_,…, *μ*_*k*_ ∈ *R^n^*) of the clusters *c*(*k*), and then the distance of sample *x*(*i*) from the nearest center point *μ*(*j*) was calculated, as shown in Formula (1). Based on the Lovett scale, during the experiment the cluster *k* was assigned a value of six

(1) $$c\left(j\right)={\mathrm{argmin}}_{j}{\left\|x(i)-\mu (j)\right\|}^{2},j\in 1,\text{…},k$$

Formula (2) was used to recalculate the value of the cluster center *μ*(*j*), where *n*_*c*(*j*)_ was the number of data in the *j*th cluster

(2) $$\mu \left(j\right)=\frac{\sum_{x\in c(j)}x_{j}}{n_{c(j)}}$$

After iterations until convergence, an accurate assortment of processed muscle strength signals is obtained.

Finally, to assess the patients' hand muscle strength status more accurately, integrating the Lovett grading standard (Table S16, Supporting Information), hand muscle strength grades (M0 to M5, where M0 indicates the worst muscle strength) were provided together with visual results (Figure S7c, Supporting Information).

### Hand Stability Assessment

To comprehensively assess hand stability, it is necessary to fuse the acceleration values measured by the IMU sensor on the three axes (i.e., *x*, *y*, and *z*). This is because interpreting acceleration data separately on each axis can be intricate and arduous. Fusing the triaxial acceleration into a single indicator can present and explain hand motion stability more conveniently. This fused indicator is commonly referred to as “combined acceleration”, calculated as follows

(3) $$a\left(k\right)=\sqrt{a_{x}{\left(k\right)}^{2}+a_{y}{\left(k\right)}^{2}+a_{z}{\left(k\right)}^{2}},k=0,1,\text{…},n-1$$

In the equation, *a*(*k*) represents the combined acceleration signal of the tremor, while *a_x_* (*k*), *a_y_* (*k*), and *a_z_* (*k*) represent acceleration signals on the *x*‐, *y*‐, and *z*‐axis, respectively. *n* represents the length of the collected acceleration data. Figure S12 (Supporting Information) shows the original triaxial acceleration signals and their combined acceleration signal. It is worth noting that the tremor signal processed subsequently refers to the combined acceleration signal obtained by fusing the acceleration signals on the three axes.

As the frequency of the static tremor mainly focuses within the range of 3–8 Hz, the sampling frequency was set as 100 Hz and the acquisition range as ±4 *g*. the signal band was then filtered around to acquire accurate tremor signals and avoid errors such as temperature drift and dark current which was introduced during data collection. Then, the median filtering (window length = 5) and bandpass filtering (cut‐off frequencies are 3 and 8 Hz) algorithms eliminated noises from systematic error, powerline interference, and human intention. Finally, Kalman Filter was adopted to remove random errors inside the signal band^[^ ^60^ ^]^ (Figure S13, Supporting Information). Specifically, based on Kalman Filter, the first two terms of the Taylor series expansion were taken from Formula (4), and Adaptive Noise Model Parameters were then added to filter errors inside the tremor signal band.

(4) $$\begin{array}{ccc} f(x) & = & \frac{f(x_{0})}{0!}+\frac{f^{\prime }\left(x_{0}\right)}{1!}\left(x-x_{0}\right)+\frac{f^{\prime \prime }\left(x_{0}\right)}{2!}{\left(x-x_{0}\right)}^{2}+\cdots +\frac{f^{\left(n\right)}\left(x_{0}\right)}{n!}{\left(x-x_{0}\right)}^{n}\\ & & +\;R_{n}\left(x\right) \end{array}$$

According to the specificity of the tremors, characterizations of processed signals in both the time domain and frequency domain were extracted to improve the grading accuracy. From the time domain, the peak‐to‐peak value, logarithmic peak‐to‐peak value, standard deviation, logarithmic standard deviation, the root‐mean‐square (RMS), and the logarithmic RMS of the triaxial acceleration were extracted. While, the frequencies of the *x*‐, *y*‐, and *z*‐axis and the combined frequency were extracted from the frequency domain. Then, the tremor signals based on the machine‐learning model were identified and classified and tenfold cross‐validation was used to determine the optimal hyperparameters of the model to avoid underfitting or overfitting problems of the model. Compared with the original UPDRS grades (0 to 4, where 4 indicates the worst stability), 0 was divided as “No tremor”, 1 and 2 as “Mild tremor”, and 3 and 4 as “Severe tremor”. The visualized grading results were also displayed on the HCI interface (Figure S7d, Supporting Information).

### Statistical Analysis

To verify the consistency between the multimodal sensor glove system and clinical assessment, the Kappa consistency test model was used. The Kappa coefficient value determines the degree of consistency, which is classified as excellent (0.8 < Kappa ≤ 1.0), good (0.6 < Kappa ≤ 0.8), moderate (0.4 < Kappa ≤ 0.6), or poor otherwise. Z‐score and *P*‐value were used to test for significant consistency in the Kappa coefficient. If the Z‐score is greater than 1.96 or less than −1.96, and the *P*‐value is less than the significance level, there is significant consistency. The sample size for finger flexibility and hand muscle strength is the same (*n* = 12), while the samples for hand stability assessment were divided into a training set and test set in an 8:2 ratio. A consistency analysis of predictions was conducted on the test set (*n* = 24) with doctor's assessments.

Furthermore, the ICC consistency model was employed to determine the test‐retest reliability of the multimodal sensor glove. Reliability was classified as excellent (ICC > 0.9), good (0.75 < ICC ≤ 0.9), moderate (0.5 < ICC ≤ 0.75), or poor otherwise. Through three repeated measurement reliability experiments, the ICC values and 95% confidence intervals were calculated for the results of 10 hand function assessments performed by 12 subjects, and comprehensively validated the reliability of the multimodal sensor glove for assessing hand dysfunctions. The Kappa and ICC consistency tests were conducted using IBM SPSS Statistics software (Version 26.0).

## Conflict of Interest

The authors declare no conflict of interest.

## Supporting information

Supporting Information

Supplemental Video 1

## Data Availability

The data that support the findings of this study are available from the corresponding author upon reasonable request.
