# Supplementary material for: Learning Hand Kinematics for Parkinson's Disease Assessment Using a Multimodal Sensor Glove
Source: Adv Sci (Weinh). 2023 May 7;10(20):2206982. doi: 10.1002/advs.202206982 (PMC10369271; doi:10.1002/advs.202206982)
Supplement: Supplementary file 1 — Supporting Information [file ADVS-10-2206982-s001.pdf]

ADVANCED SCIENCE
Open Access

# Supporting Information

for *Adv. Sci.*, DOI 10.1002/advs.202206982

Learning Hand Kinematics for Parkinson's Disease Assessment Using a Multimodal Sensor Glove

*Yu Li, Junyi Yin, Shuoyan Liu, Bing Xue, Cyrus Shokoohi, Gang Ge, Menglei Hu, Tenghuan Li, Xue Tao, Zhi Rao, Fanye Meng, Hongfeng Shi, Xiaoqiang Ji*, Peyman Servati, Xiao Xiao* and Jun Chen**

# Supplementary materials

## Learning hand kinematics for Parkinson's disease assessment using a multimodal sensor glove

*Yu Li, Junyi Yin, Shuoyan Liu, Bing Xue, Cyrus Shokoohi, Gang Ge, Menglei Hu, Tenghuan Li, Xue Tao, Zhi Rao, Fanye Meng, Hongfeng Shi, Xiaoqiang Ji*, Peyman Servati, Xiao Xiao*, Jun Chen**

Y. Li, T. Li, X. Tao, Z. Rao, F. Meng, Prof. X. Ji
School of Life Science and Technology, Changchun University of Science and Technology, Changchun 130022, China
Email: jixq2012@cust.edu.cn (X.J.)

J. Yin, X. Xiao, Prof. J. Chen
Department of Bioengineering, University of California, Los Angeles, Los Angeles, California 90095, United States
Email: jun.chen@ucla.edu (J.C.)

S. Liu, B. Xue
Department of Materials Science and Engineering, National University of Singapore, Singapore 117583, Singapore

C. Shokoohi
College of Osteopathic Medicine, Touro University Nevada, Henderson, Nevada 89014, United States

M. Hu, Prof. P. Servati
Department of Electrical and Computer Engineering, University of British Columbia, Vancouver, V6T1Z4, Canada

Dr. H. Shi
China Japan Union Hospital of Jilin University, Changchun 130033, China

G. Ge, X. Xiao
Department of Electrical and Computer Engineering, National University of Singapore, Singapore, 117583, Singapore.
Email: xiao.xiao@u.nus.edu (X.X.)

Prof. J. Chen
SKKU Institute of Energy Science and Technology, Sungkyunkwan University, Suwon 16419, Republic of Korea.

## Table of Contents

**Supplementary materials** ........ 1
Table S1: Comparison of our proposed solution with the methods suggested in previous literature ........ 3
Table S2: Comparison of finger bending angles in the patient and the healthy subject ........ 4
Table S3: Comparison of finger flexibility assessment ........ 5
Table S4: Hand muscle strength values for subjects with four actions ........ 6
Table S5: Comparison of hand muscle strength assessment ........ 7
Table S6: The characteristic values of the tremor signal and the label marked by the doctor ........ 8
Table S7: Accuracy comparison of different hidden nodes of BPNN ........ 11
Table S8: Accuracy comparison of different regularization parameters of SVM ........ 12
Table S9: Accuracy comparison of different n_neighbors of KNN ........ 13
Table S10: Accuracy comparison of different max_depth of DT ........ 14
Table S11: Accuracy of different machine learning classifiers in tremor recognition ........ 15
Table S12: Test-retest reliability results ........ 16
Table S13: Characteristics of subjects ........ 17
Table S14: Bending angle range and its corresponding grade ........ 18
Table S15: TAM assessment standard ........ 19
Table S16: Lovett grading standard ........ 20
Figure S1: Main control circuit board (43mm*38mm) ........ 21
Figure S2: STM32F103C8T6 controller minimum system ........ 22
Figure S3: The circuit schematic of the bending sensor module ........ 23
Figure S4: The circuit schematic of the flexible thin-film pressure sensor module ........ 24
Figure S5: Pin configuration and peripheral circuit diagram of MPU-9250 ........ 25
Figure S6: The circuit schematic of the power supply module ........ 26
Figure S7: Schematic diagram of the HCI interface (a) Main interface; (b) Finger flexibility assessment interface, which is used to display the bending angle of five fingers corresponding to the completion of each gesture, the maximum bending value of five fingers, and the result of flexibility assessment; (c) Hand muscle strength assessment interface, which is used to display the completed muscle strength signal for each action, the maximum value of muscle strength, and the result of muscle strength assessment; (d) Hand stability assessment interface, which is used to display the acceleration signal of hand tremor and the stability assessment result. ........ 27
Figure S8: Comparison of the accuracy of nodes in different hidden ........ 28
Figure S9: Confusion matrix of tremor recognizer ........ 29
Figure S10: Original and filtered bending signals ........ 30
Figure S11: Original and filtered muscle strength signals ........ 31
Figure S12: Triaxial acceleration and combined acceleration of tremor signals ........ 32
Figure S13: Triaxial original acceleration signals and filtered tremor signals ........ 33

**Table S1: Comparison of our proposed solution with the methods suggested in previous literature**

| | Target signals | Sensor Type | Accuracy | Statistical Analysis |
|---|---|---|---|---|
| Y Zheng et al.[1] | Joint angle, Force exerted by the finger on an object | Resistive bending sensor and resistive force sensor | Angle error: ±6°; Force error: ±8g | ICC of the bending sensor: 0.9561±0.0431, ICC of the force sensor: 0.9876±0.0058 |
| S Jiang et al.[2] | Air gestures, surface gestures | Surface electromyography and IMU sensing fusion | Air gestures: 92.6% (LDA), surface gestures: 88.8% (LDA) | – |
| HB Kim et al.[3] | Tremor | Accelerometer and gyroscope | 0.85(CNN) | Kappa value of the system assessment and the doctor’s assessment: 0.85 |
| Q Fang et al.[4] | Joint angle | Infrared imaging sensor | Percentage residual between the system measurements and protractor measurements: 8.8% | CC between the system measurements and protractor measurements: 0.9672 |
| Y Zou et al.[5] | 15 categories of activities, nonstandard behaviors | IMU | Higher than 95% (Half-DTW) | – |
| X Chen et al.[6] | Finger gestures, task-oriented gestures | Flexion sensor, force sensor | Finger gestures: 93.2% (SVM), task-oriented gestures: 89.4% (SVM) | – |
| Our multimodal sensor glove | Rigidity, muscle strength, tremor | Flexible bending sensors, flexible thin-film pressure sensors, and IMU sensors | Stability assessment: 95.83% （BPNN） | Kappa value for flexibility assessment: 0.833, Kappa value for muscle strength assessment: 0.867, Kappa value for stability assessment: 0.937 |

## References


[1] Y. Zheng, Y. Peng, G. Wang, X. Liu, X. Dong, J. Wang, *Measurement*. **2016**, *93*, 1.

[2] S. Jiang, B. Lv, W. Guo, C. Zhang, H. Wang, X. Sheng, P. B. Shull, *IEEE Trans Industr Inform*. **2017**, *14*, 3376.

[3] H. B. Kim, W. W. Lee, A. Kim, H. J. Lee, H. Y. Park, H. S. Jeon, S. K. Kim, B. Jeon, K. S. Park, *Comput. Biol. Med*. **2018**, *95*, 140.

[4] Q. Fang, S. S. Mahmoud, X. Gu, J. Fu, *IEEE J Biomed Health Inform*. **2018**, *23*, 758.

[5] Y. Zou, D. Wang, S. Hong, R. Ruby, D. Zhang, K. Wu, *IEEE Internet Things J.* **2020**, *7*, 7377.

[6] X. Chen, L. Gong, L. Wei, S.-C. Yeh, L. Da Xu, L. Zheng, Z. Zou, *IEEE Trans Industr Inform.* **2020**, *17*, 943.

**Table S2: Comparison of finger bending angles in the patient and the healthy subject**

| Gestures | Subjects | Thumb angle (°) | Index angle (°) | Middle angle (°) | Ring angle (°) | Little angle (°) |
|---|---|---|---|---|---|---|
| Flat hand | Healthy subject | 0 | 0.102 | 0.206 | 0.995 | 0.716 |
| | Patient | 9.201 | 11.204 | 10.524 | 9.236 | 12.478 |
| Fist | Healthy subject | 99.82 | 119.594 | 108.71 | 96.04 | 91.784 |
| | Patient | 58.358 | 59.458 | 63.003 | 61.256 | 57.859 |
| OK | Healthy subject | 91.34 | 109.525 | 0.308 | 0.915 | 0.721 |
| | Patient | 56.118 | 58.437 | 11.002 | 12.315 | 10.261 |
| Orchid fingers | Healthy subject | 91.68 | 0.442 | 108.44 | 1.562 | 0.893 |
| | Patient | 71.012 | 40.501 | 75.021 | 30.251 | 11.416 |
| A | Healthy subject | 98.92 | 0.21 | 0.103 | 119.04 | 0.756 |
| | Patient | 65.241 | 27.21 | 18.231 | 66.216 | 45.318 |
| W | Healthy subject | 97.71 | 0.21 | 0.214 | 1.097 | 93.812 |
| | Patient | 66.311 | 10.12 | 11.021 | 10.23 | 68.448 |
| B | Healthy subject | 97.77 | 112.619 | 113.57 | 1.69 | 0.745 |
| | Patient | 68.354 | 65.256 | 60.231 | 10.256 | 10.118 |
| U | Healthy subject | 98.36 | 0.223 | 105.02 | 110.96 | 0.745 |
| | Patient | 68.369 | 10.249 | 65.231 | 60.256 | 10.346 |
| V | Healthy subject | 0.03 | 116.802 | 108.63 | 1.089 | 0.727 |
| | Patient | 68.003 | 10.562 | 10.49 | 65.264 | 65.361 |

**Table S3: Comparison of finger flexibility assessment**

| Subject No. | Thumb angle (°) | Index angle (°) | Middle angle (°) | Ring angle (°) | Little angle (°) | System | Doctor |
|---|---|---|---|---|---|---|---|
| 1 | 99.820 | 119.59 | 108.71 | 96.040 | 91.784 | F4 | F4 |
| 2 | 98.256 | 116.70 | 105.26 | 93.210 | 91.006 | F4 | F4 |
| 3 | 99.315 | 118.97 | 107.56 | 95.246 | 91.231 | F4 | F4 |
| 4 | 100.20 | 119.69 | 109.79 | 99.650 | 95.264 | F4 | F4 |
| 5 | 99.213 | 118.66 | 107.25 | 95.001 | 91.198 | F4 | F4 |
| 6 | 98.765 | 117.86 | 106.56 | 94.521 | 91.018 | F4 | F4 |
| 7 | 99.896 | 119.60 | 108.65 | 95.346 | 94.267 | F4 | F4 |
| 8 | 100.06 | 119.63 | 109.00 | 98.796 | 95.012 | F4 | F4 |
| 9 | 58.358 | 59.458 | 63.003 | 61.256 | 57.259 | F2 | F2 |
| 10 | 65.269 | 70.015 | 70.280 | 69.315 | 68.457 | F3 | F3 |
| 11 | 63.310 | 65.214 | 65.216 | 63.256 | 59.009 | F3 | F2 |
| 12 | 65.104 | 68.265 | 68.214 | 69.003 | 63.466 | F3 | F3 |

**Table S4: Hand muscle strength values for subjects with four actions**

| Subject No. | Grasp the cylinder(N) | Pinch objects with fingertip(N) | Grasp the ball(N) | Click objects with single finger(N) |
|---|---|---|---|---|
| 1 | 14.545 | 6.362 | 9.726 | 3.551 |
| 2 | 15.314 | 5.944 | 9.673 | 2.886 |
| 3 | 15.128 | 5.665 | 8.738 | 3.023 |
| 4 | 13.247 | 6.841 | 9.407 | 2.715 |
| 5 | 16.298 | 7.681 | 9.002 | 2.986 |
| 6 | 15.155 | 6.414 | 8.968 | 2.641 |
| 7 | 14.968 | 6.710 | 10.087 | 3.101 |
| 8 | 13.630 | 6.633 | 8.988 | 2.885 |
| 9 | 0 | 1.392 | 2.205 | 1.282 |
| 10 | 6.378 | 2.083 | 4.629 | 1.382 |
| 11 | 6.084 | 2.713 | 4.332 | 1.158 |
| 12 | 7.157 | 1.879 | 3.689 | 1.136 |

**Table S5: Comparison of hand muscle strength assessment**

| Subject No. | Muscle strength value (N) | System | Doctor |
|---|---|---|---|
| 1 | 14.55 | M4 | M4 |
| 2 | 15.31 | M4 | M5 |
| 3 | 15.13 | M4 | M4 |
| 4 | 13.25 | M4 | M4 |
| 5 | 16.30 | M5 | M5 |
| 6 | 15.16 | M4 | M4 |
| 7 | 14.97 | M4 | M4 |
| 8 | 13.63 | M4 | M4 |
| 9 | 0.000 | M0 | M0 |
| 10 | 6.378 | M2 | M2 |
| 11 | 6.084 | M2 | M2 |
| 12 | 7.157 | M2 | M2 |

**Table S6: The characteristic values of the tremor signal and the label marked by the doctor**

| a(1) | $ln(a)$(2) | $rms$(3) | $ln(rms)$(4) | $vpp$(5) | $ln(vpp)$(6) | $std$(7) | $ln(std)$(8) | $p_f$(9) | Label |
|---|---|---|---|---|---|---|---|---|---|
| 0.000030 | -2.851460 | 0.014771 | -2.728709 | 0.014208 | -2.756784 | 0.005752 | -3.145751 | 0.017250 | 0 |
| 0.000051 | -2.388829 | 0.016548 | -2.248595 | 0.017070 | -2.196049 | 0.006928 | -2.584288 | 0.016813 | 0 |
| 0.000035 | -2.277593 | 0.017278 | -2.156877 | 0.017223 | -2.174182 | 0.006986 | -2.563832 | 0.016013 | 0 |
| 0.000053 | -2.285369 | 0.016774 | -2.167150 | 0.016958 | -2.165231 | 0.006879 | -2.555175 | 0.016168 | 0 |
| 0.000047 | -2.428037 | 0.015901 | -2.298972 | 0.016120 | -2.287763 | 0.006542 | -2.677084 | 0.016154 | 0 |
| 0.000041 | -2.398392 | 0.015794 | -2.284112 | 0.015138 | -2.362148 | 0.006131 | -2.752495 | 0.017380 | 0 |
| 0.000032 | -2.398243 | 0.015853 | -2.271495 | 0.016255 | -2.238782 | 0.006595 | -2.627639 | 0.016612 | 0 |
| 0.000084 | -2.318580 | 0.017160 | -2.189021 | 0.017281 | -2.176795 | 0.007009 | -2.565643 | 0.018068 | 0 |
| 0.000057 | -2.540110 | 0.015212 | -2.426212 | 0.014831 | -2.448581 | 0.006012 | -2.837852 | 0.017089 | 0 |
| 0.000107 | -2.344838 | 0.016394 | -2.217771 | 0.016563 | -2.213056 | 0.006720 | -2.601686 | 0.015929 | 0 |
| -0.000005 | -2.391808 | 0.016029 | -2.275982 | 0.015839 | -2.306146 | 0.006418 | -2.696387 | 0.016860 | 0 |
| 0.000041 | -2.429266 | 0.016106 | -2.301727 | 0.015540 | -2.330316 | 0.006291 | -2.719743 | 0.017626 | 0 |
| 0.000035 | -2.836316 | 0.014126 | -2.719394 | 0.013702 | -2.737093 | 0.005549 | -3.126851 | 0.016231 | 0 |
| 0.000048 | -2.762336 | 0.014200 | -2.652019 | 0.013637 | -2.710195 | 0.005520 | -3.100506 | 0.016078 | 0 |
| 0.000039 | -2.675288 | 0.014629 | -2.551089 | 0.014205 | -2.545826 | 0.005751 | -2.935958 | 0.016540 | 0 |
| 0.000043 | -2.484796 | 0.016159 | -2.355946 | 0.016277 | -2.311052 | 0.006602 | -2.699182 | 0.017159 | 0 |
| 0.000055 | -2.319327 | 0.017051 | -2.200401 | 0.017054 | -2.197961 | 0.006915 | -2.586961 | 0.017654 | 0 |
| 0.000011 | -2.382678 | 0.015941 | -2.255737 | 0.016119 | -2.256085 | 0.006540 | -2.643782 | 0.016320 | 0 |
| 0.000037 | -2.469259 | 0.015326 | -2.350423 | 0.015107 | -2.375412 | 0.006125 | -2.763520 | 0.017399 | 0 |
| 0.000083 | -2.379932 | 0.016481 | -2.248461 | 0.017047 | -2.208229 | 0.006919 | -2.597218 | 0.016429 | 0 |
| 0.000032 | -2.775351 | 0.016366 | -2.651739 | 0.015681 | -2.710770 | 0.006346 | -3.102606 | 0.019037 | 0 |
| 0.000033 | -2.785642 | 0.014999 | -2.672113 | 0.014389 | -2.733629 | 0.005824 | -3.124401 | 0.017176 | 0 |
| 0.000036 | -2.552982 | 0.015298 | -2.439429 | 0.015240 | -2.465420 | 0.006182 | -2.854222 | 0.017580 | 0 |
| 0.000028 | -2.550884 | 0.015621 | -2.434582 | 0.015299 | -2.454259 | 0.006201 | -2.843045 | 0.016566 | 0 |
| 0.000058 | -2.680944 | 0.014696 | -2.551476 | 0.014212 | -2.549412 | 0.005755 | -2.939373 | 0.016277 | 0 |
| 0.000052 | -2.813203 | 0.014239 | -2.698929 | 0.013776 | -2.717771 | 0.005579 | -3.106857 | 0.016296 | 0 |
| 0.000053 | -2.891002 | 0.014165 | -2.761601 | 0.013612 | -2.760141 | 0.005508 | -3.150368 | 0.016346 | 0 |
| 0.000052 | -2.780897 | 0.014253 | -2.666676 | 0.013943 | -2.664214 | 0.005648 | -3.052718 | 0.016326 | 0 |
| 0.000037 | -2.824247 | 0.014239 | -2.708627 | 0.013749 | -2.695809 | 0.005567 | -3.085409 | 0.016412 | 0 |
| 0.000046 | -2.772028 | 0.014247 | -2.649865 | 0.013777 | -2.667984 | 0.005578 | -3.057760 | 0.016256 | 0 |
| 0.000042 | -2.781325 | 0.014261 | -2.664831 | 0.013789 | -2.703047 | 0.005583 | -3.093299 | 0.016505 | 0 |
| 0.000044 | -2.735171 | 0.014438 | -2.610913 | 0.013955 | -2.624112 | 0.005648 | -3.013633 | 0.016107 | 0 |
| 0.000049 | -2.738475 | 0.014673 | -2.623980 | 0.014080 | -2.672639 | 0.005703 | -3.061443 | 0.016261 | 0 |
| 0.000037 | -2.802050 | 0.014303 | -2.677647 | 0.013852 | -2.664269 | 0.005609 | -3.053889 | 0.016289 | 0 |
| 0.000039 | -2.857273 | 0.014076 | -2.732692 | 0.013589 | -2.737778 | 0.005501 | -3.127077 | 0.016768 | 0 |
| 0.000031 | -2.816360 | 0.014121 | -2.703158 | 0.013613 | -2.721710 | 0.005510 | -3.111796 | 0.016226 | 0 |
| 0.000036 | -2.817512 | 0.014209 | -2.691561 | 0.013765 | -2.690475 | 0.005571 | -3.080044 | 0.016181 | 0 |
| 0.000045 | -2.869202 | 0.014064 | -2.741503 | 0.013537 | -2.771172 | 0.005480 | -3.160949 | 0.016256 | 0 |
| 0.000041 | -2.710718 | 0.014377 | -2.588689 | 0.014088 | -2.591960 | 0.005706 | -2.981536 | 0.015847 | 0 |
| 0.000036 | -2.845144 | 0.014062 | -2.726375 | 0.013581 | -2.736607 | 0.005498 | -3.126473 | 0.016457 | 0 |
| -0.000204 | -1.735241 | 0.032555 | -1.628874 | 0.027314 | -1.781679 | 0.010992 | -2.175945 | 0.039846 | 1 |

| | | | | | | | | | |
|---|---|---|---|---|---|---|---|---|---|
| 0.000072 | -1.886117 | 0.026849 | -1.731617 | 0.031577 | -1.657038 | 0.012860 | -2.044945 | 0.018241 | 1 |
| 0.000218 | -1.583838 | 0.041183 | -1.440282 | 0.050677 | -1.367102 | 0.020648 | -1.755353 | 0.035779 | 1 |
| 0.000076 | -1.639157 | 0.038222 | -1.499326 | 0.048527 | -1.409675 | 0.019794 | -1.797342 | 0.037838 | 1 |
| 0.000123 | -1.738147 | 0.031599 | -1.604942 | 0.037924 | -1.542365 | 0.015448 | -1.930468 | 0.026465 | 1 |
| -0.000086 | -1.706462 | 0.032821 | -1.567551 | 0.040806 | -1.479591 | 0.016638 | -1.867695 | 0.029723 | 1 |
| -0.000665 | -1.343335 | 0.078105 | -1.177142 | 0.114248 | -1.029918 | 0.046950 | -1.415326 | 0.056216 | 1 |
| 0.000247 | -1.782099 | 0.030046 | -1.637634 | 0.035626 | -1.576912 | 0.014506 | -1.964963 | 0.032262 | 1 |
| 0.000017 | -2.094378 | 0.020649 | -1.970181 | 0.022494 | -1.922712 | 0.009145 | -2.309985 | 0.017420 | 1 |
| 0.000143 | -2.211599 | 0.018918 | -2.084005 | 0.019278 | -2.086103 | 0.007816 | -2.474801 | 0.018094 | 1 |
| 0.000026 | -1.606480 | 0.043034 | -1.451607 | 0.058720 | -1.330036 | 0.024031 | -1.716940 | 0.034651 | 1 |
| 0.000206 | -1.477268 | 0.055873 | -1.339304 | 0.072696 | -1.257490 | 0.029694 | -1.644709 | 0.039659 | 1 |
| -0.000029 | -1.807884 | 0.030675 | -1.665985 | 0.036669 | -1.589519 | 0.014936 | -1.977142 | 0.023076 | 1 |
| 0.000127 | -1.907569 | 0.027470 | -1.783427 | 0.031626 | -1.735886 | 0.012898 | -2.123264 | 0.022493 | 1 |
| -0.000192 | -1.542351 | 0.044557 | -1.402995 | 0.057939 | -1.302514 | 0.023645 | -1.689817 | 0.035314 | 1 |
| 0.000008 | -1.509611 | 0.048644 | -1.368240 | 0.065029 | -1.256074 | 0.026584 | -1.643033 | 0.051031 | 1 |
| 0.000061 | -1.939152 | 0.024556 | -1.800439 | 0.028266 | -1.722572 | 0.011514 | -2.110215 | 0.018816 | 1 |
| 0.000033 | -1.830624 | 0.029284 | -1.694288 | 0.036032 | -1.605504 | 0.014708 | -1.992794 | 0.023035 | 1 |
| -0.000122 | -1.662223 | 0.038544 | -1.511780 | 0.050165 | -1.397863 | 0.020500 | -1.784845 | 0.034508 | 1 |
| -0.000312 | -1.738935 | 0.033590 | -1.591210 | 0.040251 | -1.517627 | 0.016395 | -1.905748 | 0.021220 | 1 |
| -0.000153 | -1.570424 | 0.043601 | -1.437902 | 0.048646 | -1.404560 | 0.019714 | -1.794319 | 0.029529 | 1 |
| -0.000405 | -1.494693 | 0.050449 | -1.345133 | 0.065193 | -1.249335 | 0.026589 | -1.637567 | 0.036261 | 1 |
| 0.000039 | -1.758848 | 0.034536 | -1.629576 | 0.040060 | -1.583411 | 0.016296 | -1.972533 | 0.023599 | 1 |
| -0.000120 | -1.681239 | 0.042023 | -1.511678 | 0.056930 | -1.359061 | 0.023400 | -1.743038 | 0.026030 | 1 |
| -0.000074 | -1.482161 | 0.058352 | -1.345097 | 0.067249 | -1.290992 | 0.027471 | -1.678489 | 0.034829 | 1 |
| -0.000029 | -1.549027 | 0.051722 | -1.388533 | 0.070123 | -1.267165 | 0.028756 | -1.653387 | 0.036362 | 1 |
| 0.000617 | -1.727937 | 0.038487 | -1.586522 | 0.043608 | -1.534745 | 0.017766 | -1.922574 | 0.030985 | 1 |
| 0.000039 | -2.167997 | 0.019191 | -2.040183 | 0.020508 | -1.995110 | 0.008323 | -2.384089 | 0.016820 | 1 |
| 0.000046 | -2.122838 | 0.020608 | -2.000246 | 0.022378 | -1.957912 | 0.009089 | -2.346857 | 0.017904 | 1 |
| 0.000108 | -1.606689 | 0.046738 | -1.484796 | 0.053395 | -1.454635 | 0.021784 | -1.842782 | 0.034662 | 1 |
| 0.000127 | -1.841413 | 0.033348 | -1.681491 | 0.041616 | -1.576958 | 0.017053 | -1.962154 | 0.025601 | 1 |
| 0.002083 | -0.957498 | 0.163587 | -0.812986 | 0.240718 | -0.674575 | 0.098656 | -1.061462 | 0.165630 | 1 |
| 0.000053 | -1.746744 | 0.034033 | -1.606566 | 0.043002 | -1.498606 | 0.017593 | -1.884586 | 0.022290 | 1 |
| -0.000049 | -1.476414 | 0.057542 | -1.324958 | 0.079424 | -1.191703 | 0.032530 | -1.578246 | 0.029486 | 1 |
| 0.000035 | -1.854541 | 0.028817 | -1.720217 | 0.034615 | -1.646975 | 0.014126 | -2.034376 | 0.019760 | 1 |
| 0.000006 | -1.647686 | 0.040876 | -1.516319 | 0.051302 | -1.446435 | 0.020952 | -1.833978 | 0.028393 | 1 |
| 0.001095 | -0.942521 | 0.168246 | -0.798862 | 0.239950 | -0.672840 | 0.098191 | -1.059714 | 0.140198 | 1 |
| 0.000539 | -1.634031 | 0.041337 | -1.499920 | 0.050602 | -1.420032 | 0.020657 | -1.807317 | 0.022775 | 1 |
| 0.000224 | -1.358053 | 0.066277 | -1.224290 | 0.086306 | -1.141456 | 0.035217 | -1.529559 | 0.069957 | 1 |
| -0.000153 | -1.570424 | 0.043601 | -1.437902 | 0.048646 | -1.404560 | 0.019714 | -1.794319 | 0.029529 | 1 |
| -0.000092 | -1.359092 | 0.077239 | -1.211496 | 0.113131 | -1.073284 | 0.046424 | -1.459622 | 0.071284 | 1 |
| 0.001166 | -0.945565 | 0.170124 | -0.788677 | 0.251402 | -0.652242 | 0.103157 | -1.038282 | 0.166740 | 2 |
| 0.000385 | -1.007355 | 0.145736 | -0.868668 | 0.201928 | -0.754240 | 0.082491 | -1.141682 | 0.184985 | 2 |
| 0.000012 | -1.109630 | 0.117431 | -0.958858 | 0.161476 | -0.845557 | 0.065955 | -1.232891 | 0.138682 | 2 |
| 0.000592 | -1.063120 | 0.126834 | -0.922630 | 0.172868 | -0.816858 | 0.070626 | -1.204223 | 0.154794 | 2 |

| | | | | | | | | | |
|---|---|---|---|---|---|---|---|---|---|
| 0.001794 | -0.818594 | 0.228526 | -0.661465 | 0.334151 | -0.524334 | 0.136865 | -0.911033 | 0.199557 | 2 |
| -0.001910 | -0.882082 | 0.196459 | -0.733497 | 0.276439 | -0.613538 | 0.113055 | -1.000944 | 0.176885 | 2 |
| 0.000593 | -0.872548 | 0.205898 | -0.725741 | 0.306751 | -0.576797 | 0.125869 | -0.962689 | 0.194082 | 2 |
| -0.001401 | -0.701288 | 0.295029 | -0.559685 | 0.452331 | -0.396394 | 0.185816 | -0.782041 | 0.228946 | 2 |
| 0.000642 | -0.915543 | 0.184591 | -0.766715 | 0.264227 | -0.644124 | 0.108172 | -1.031128 | 0.132823 | 2 |
| -0.003182 | -0.537952 | 0.442488 | -0.386337 | 0.665440 | -0.246179 | 0.273252 | -0.631860 | 0.378825 | 2 |
| -0.000872 | -0.886904 | 0.203630 | -0.728595 | 0.309772 | -0.566248 | 0.127270 | -0.951913 | 0.214813 | 2 |
| 0.000696 | -0.882855 | 0.193163 | -0.733213 | 0.269058 | -0.619079 | 0.109929 | -1.006378 | 0.148794 | 2 |
| -0.002763 | -0.846441 | 0.238665 | -0.695585 | 0.342486 | -0.584121 | 0.140658 | -0.970181 | 0.187718 | 2 |
| -0.000579 | -0.853602 | 0.226709 | -0.689817 | 0.329399 | -0.555968 | 0.135111 | -0.941712 | 0.166269 | 2 |
| -0.000724 | -0.896175 | 0.197206 | -0.744546 | 0.262492 | -0.657832 | 0.107161 | -1.045981 | 0.119658 | 2 |
| -0.000040 | -0.534188 | 0.433236 | -0.380491 | 0.626277 | -0.250102 | 0.256373 | -0.637094 | 0.339063 | 2 |
| 0.001917 | -0.595476 | 0.386157 | -0.448812 | 0.543837 | -0.335482 | 0.222348 | -0.722980 | 0.321190 | 2 |
| 0.001371 | -0.627690 | 0.352234 | -0.475877 | 0.514055 | -0.339936 | 0.210650 | -0.726037 | 0.287621 | 2 |
| 0.001895 | -0.751664 | 0.275885 | -0.608040 | 0.396202 | -0.482015 | 0.162193 | -0.868854 | 0.339640 | 2 |
| 0.001428 | -0.590262 | 0.372865 | -0.445093 | 0.535798 | -0.318203 | 0.219267 | -0.705134 | 0.424548 | 2 |
| 0.000478 | -1.268975 | 0.090850 | -1.116385 | 0.125000 | -0.999525 | 0.051117 | -1.386594 | 0.069033 | 2 |
| -0.000464 | -1.153486 | 0.123047 | -0.988010 | 0.182771 | -0.833304 | 0.075091 | -1.218714 | 0.110213 | 2 |
| 0.000654 | -1.122583 | 0.115287 | -0.985098 | 0.148667 | -0.897093 | 0.060568 | -1.285023 | 0.119635 | 2 |
| -0.000796 | -1.046313 | 0.133326 | -0.903195 | 0.187198 | -0.783970 | 0.076588 | -1.170798 | 0.111983 | 2 |
| -0.000024 | -1.550838 | 0.052052 | -1.403924 | 0.055614 | -1.385638 | 0.022667 | -1.773089 | 0.043046 | 2 |
| 0.000413 | -1.546136 | 0.048901 | -1.408424 | 0.061916 | -1.327390 | 0.025366 | -1.715115 | 0.033221 | 2 |
| -0.000916 | -0.704276 | 0.310608 | -0.538607 | 0.455427 | -0.394705 | 0.186656 | -0.780599 | 0.236163 | 2 |
| -0.000062 | -1.175426 | 0.105904 | -1.026974 | 0.163662 | -0.852069 | 0.067350 | -1.236421 | 0.080852 | 2 |
| 0.002526 | -0.848777 | 0.213420 | -0.718451 | 0.248387 | -0.703294 | 0.100976 | -1.093583 | 0.115091 | 2 |
| 0.001240 | -0.846402 | 0.211228 | -0.701166 | 0.288379 | -0.596985 | 0.117773 | -0.984618 | 0.168719 | 2 |
| 0.000275 | -1.034104 | 0.166852 | -0.869941 | 0.239357 | -0.715305 | 0.098265 | -1.100812 | 0.098139 | 2 |
| -0.000289 | -1.184592 | 0.096638 | -1.037350 | 0.139143 | -0.905971 | 0.056980 | -1.292759 | 0.085401 | 2 |
| -0.002375 | -0.725114 | 0.286735 | -0.569395 | 0.437185 | -0.407608 | 0.179629 | -0.792936 | 0.210797 | 2 |
| -0.000922 | -0.948713 | 0.189199 | -0.794484 | 0.276130 | -0.656192 | 0.113303 | -1.041959 | 0.126189 | 2 |
| -0.000383 | -0.746858 | 0.266343 | -0.596237 | 0.398758 | -0.450635 | 0.163595 | -0.836540 | 0.227754 | 2 |
| 0.000044 | -0.971163 | 0.166932 | -0.821824 | 0.249416 | -0.672636 | 0.102419 | -1.058787 | 0.164558 | 2 |
| 0.000618 | -0.685153 | 0.292631 | -0.551013 | 0.402944 | -0.444153 | 0.164626 | -0.831553 | 0.352874 | 2 |
| -0.000504 | -1.003527 | 0.163034 | -0.856520 | 0.221897 | -0.744072 | 0.090752 | -1.130924 | 0.116638 | 2 |
| 0.000020 | -1.178877 | 0.104378 | -1.049516 | 0.123761 | -1.011020 | 0.050419 | -1.399462 | 0.057851 | 2 |
| -0.000147 | -1.071943 | 0.124826 | -0.930813 | 0.166172 | -0.843210 | 0.067835 | -1.231088 | 0.097816 | 2 |

(1) Acceleration; (2) logarithmic acceleration; (3) RMS; (4) logarithmic RMS; (5) peak-to-peak value; (6) logarithmic peak-to-peak value; (7) standard deviation; (8) logarithmic standard deviation; (9) main peak value in frequency domain.

**Table S7: Accuracy comparison of different hidden nodes of BPNN**

| Number of hidden nodes | Iterations | Average accuracy |
|---|---|---|
| 0 | 1000 | 0.69778 |
| 1 | 1000 | 0.87778 |
| 2 | 1000 | 0.90667 |
| 3 | 1000 | 0.93778 |
| 4 | 1000 | 0.91556 |
| 5 | 1000 | 0.91556 |
| **6** | **1000** | **0.94778** |
| 7 | 1000 | 0.92667 |
| 8 | 1000 | 0.93778 |
| 9 | 1000 | 0.91556 |
| 10 | 1000 | 0.92556 |
| 11 | 1000 | 0.92667 |
| 12 | 1000 | 0.92667 |
| 13 | 1000 | 0.91556 |
| 14 | 1000 | 0.91556 |
| 15 | 1000 | 0.91556 |
| 16 | 1000 | 0.91556 |
| 17 | 1000 | 0.93667 |
| 18 | 1000 | 0.91556 |
| 19 | 1000 | 0.92667 |
| 20 | 1000 | 0.92556 |
| 21 | 1000 | 0.93667 |
| 22 | 1000 | 0.92556 |
| 23 | 1000 | 0.93667 |
| 24 | 1000 | 0.91556 |
| 25 | 1000 | 0.92667 |
| 26 | 1000 | 0.93667 |
| 27 | 1000 | 0.92667 |
| 28 | 1000 | 0.93667 |
| 29 | 1000 | 0.93667 |

**Table S8: Accuracy comparison of different regularization parameters of SVM**

| **Regularization parameter** | **Average accuracy** |
|---|---|
| 0 | 0.917777778 |
| 1 | 0.917777778 |
| 2 | 0.917777778 |
| 3 | 0.917777778 |
| 4 | 0.917777778 |
| 5 | 0.897777778 |
| 6 | 0.918888889 |
| **7** | **0.928888889** |
| 8 | 0.917777778 |
| 9 | 0.918888889 |
| 10 | 0.928888889 |
| 11 | 0.928888889 |
| 12 | 0.928888889 |
| 13 | 0.928888889 |
| 14 | 0.917777778 |
| 15 | 0.917777778 |
| 16 | 0.917777778 |
| 17 | 0.917777778 |
| 18 | 0.917777778 |
| 19 | 0.917777778 |
| 20 | 0.917777778 |
| 21 | 0.917777778 |
| 22 | 0.917777778 |
| 23 | 0.917777778 |
| 24 | 0.917777778 |
| 25 | 0.917777778 |
| 26 | 0.917777778 |
| 27 | 0.917777778 |
| 28 | 0.917777778 |
| 29 | 0.917777778 |

**Table S9: Accuracy comparison of different n_neighbors of KNN**

| n_neighbors | Average accuracy |
|---|---|
| 0 | 0.885555556 |
| 1 | 0.874444444 |
| **2** | **0.936666667** |
| 3 | 0.924444444 |
| 4 | 0.925555556 |
| 5 | 0.925555556 |
| 6 | 0.925555556 |
| 7 | 0.925555556 |
| 8 | 0.925555556 |
| 9 | 0.925555556 |
| 10 | 0.925555556 |
| 11 | 0.925555556 |
| 12 | 0.925555556 |
| 13 | 0.925555556 |
| 14 | 0.925555556 |
| 15 | 0.915555556 |
| 16 | 0.925555556 |
| 17 | 0.915555556 |
| 18 | 0.915555556 |
| 19 | 0.905555556 |
| 20 | 0.905555556 |
| 21 | 0.905555556 |
| 22 | 0.905555556 |
| 23 | 0.905555556 |
| 24 | 0.905555556 |
| 25 | 0.905555556 |
| 26 | 0.915555556 |
| 27 | 0.894444444 |
| 28 | 0.905555556 |
| 29 | 0.905555556 |

**Table S10: Accuracy comparison of different max_depth of DT**

| max_depth | Average accuracy |
|---|---|
| 0 | 0.636666667 |
| **1** | **0.925555556** |
| 2 | 0.904444444 |
| 3 | 0.894444444 |
| 4 | 0.874444444 |
| 5 | 0.894444444 |
| 6 | 0.894444444 |
| 7 | 0.894444444 |
| 8 | 0.904444444 |
| 9 | 0.894444444 |
| 10 | 0.894444444 |
| 11 | 0.894444444 |
| 12 | 0.884444444 |
| 13 | 0.894444444 |
| 14 | 0.884444444 |
| 15 | 0.894444444 |
| 16 | 0.904444444 |
| 17 | 0.894444444 |
| 18 | 0.894444444 |
| 19 | 0.884444444 |
| 20 | 0.884444444 |
| 21 | 0.894444444 |
| 22 | 0.894444444 |
| 23 | 0.894444444 |
| 24 | 0.884444444 |
| 25 | 0.894444444 |
| 26 | 0.894444444 |
| 27 | 0.874444444 |
| 28 | 0.904444444 |
| 29 | 0.884444444 |

**Table S11: Accuracy of different machine learning classifiers in tremor recognition**

| Machine learning classifiers | Accuracy |
|---|---|
| **BPNN** | **0.9583** |
| SVM | 0.8333 |
| KNN | 0.875 |
| DT | 0.9167 |

**Table S12: Test-retest reliability results**

| Test contents | Sample size (n) | ICC | 95%CI | |
|---|---|---|---|---|
| | | | Lower bound | Upper bound |
| Finger flexibility assessments | 12 | 0.923 | 0.849 | 0.973 |
| Hand muscle strength assessments | 12 | 0.910 | 0.824 | 0.968 |
| Hand stability assessments | 12 | 0.946 | 0.890 | 0.981 |

**Table S13: Characteristics of subjects**

| Index | Healthy subjects | PD patients |
|---|---|---|
| Number | 32 | 8 |
| Age (min / max) | 22/27 | 63/68 |
| The average age of illness (years) | — | 9±0.77 |

**Table S14: Bending angle range and its corresponding grade**

| **Angel range** | **Normalization** | **Grade** |
|---|---|---|
| 0-10° (<10°) | 0-0.2 | F0 |
| 10-30° (<30°) | 0.2-0.5 | F1 |
| 30-60° (<60°) | 0.5-0.8 | F2 |
| 60-90° (<90°) | 0.8-1 | F3 |
| ≥ 90° | 1 | F4 |

**Table S15: TAM assessment standard**

| Grade | Standard |
|---|---|
| Poor | TAM <50% of the normal side |
| Medium | TAM >50% of the normal side, 180-200° |
| Good | TAM >75% of the normal side, 200-220° |
| Excellent | Normal range of activity, >220° |

**Table S16: Lovett grading standard**

| Grade | Standard |
|---|---|
| M0 | No muscle contractions or any contractions. |
| M1 | Muscles shrink but joints cannot be moved. |
| M2 | Joints can move slightly but muscles cannot resist gravity. |
| M3 | Joints can move. Muscles can resist gravity but cannot resist resistance. |
| M4 | Joints can move normally and muscles can resist resistance. |
| M5 | Joints can move normally and muscles can resist strong resistance. |

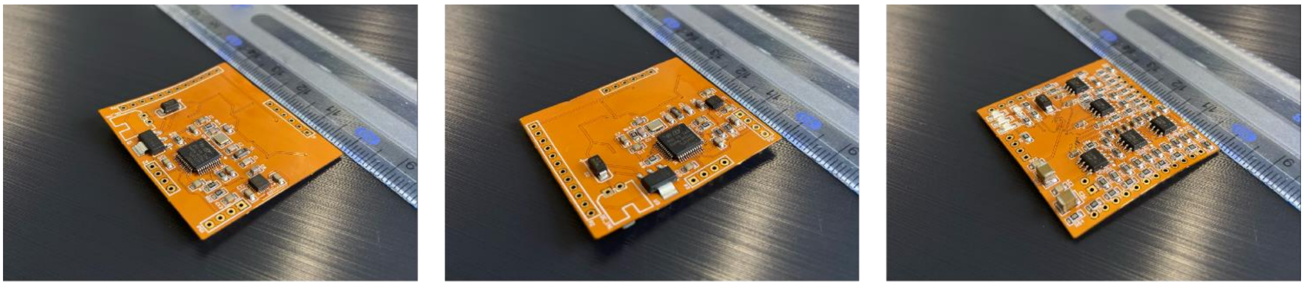

**Figure S1: Main control circuit board (43 mm*38 mm)**

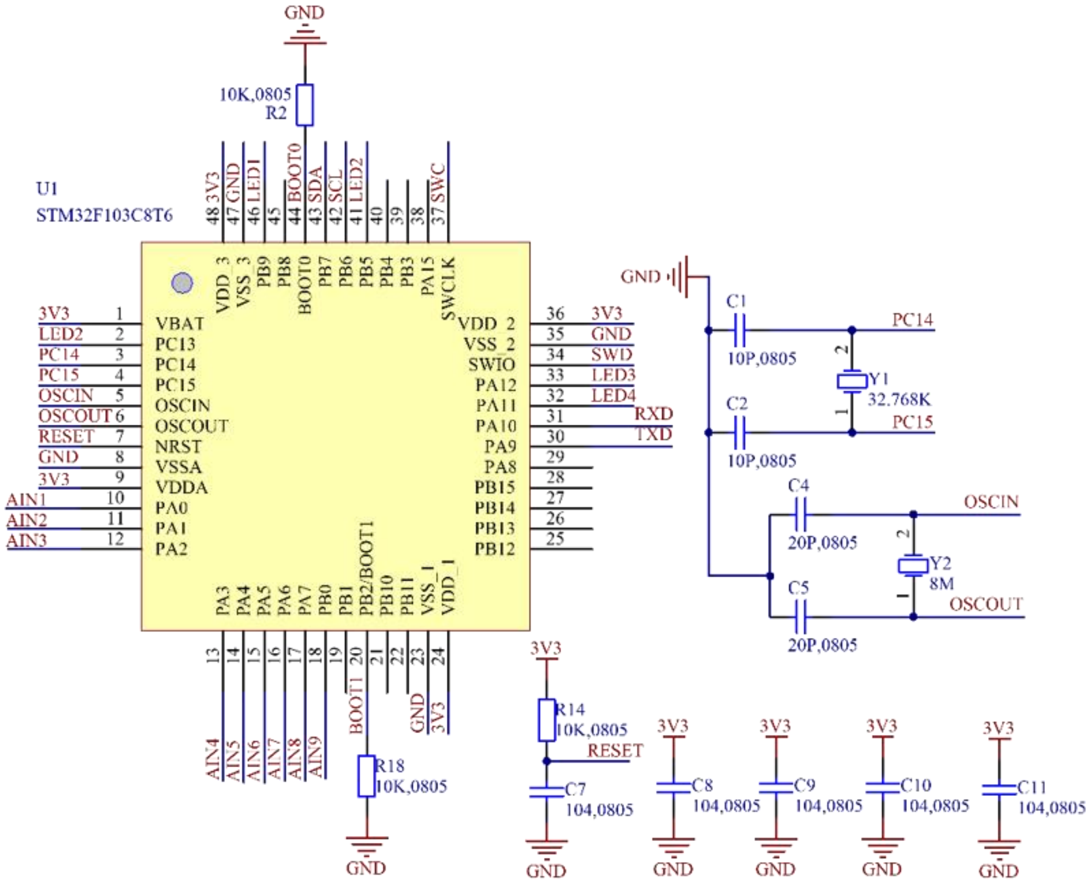


**Figure S2: STM32F103C8T6 controller minimum system**

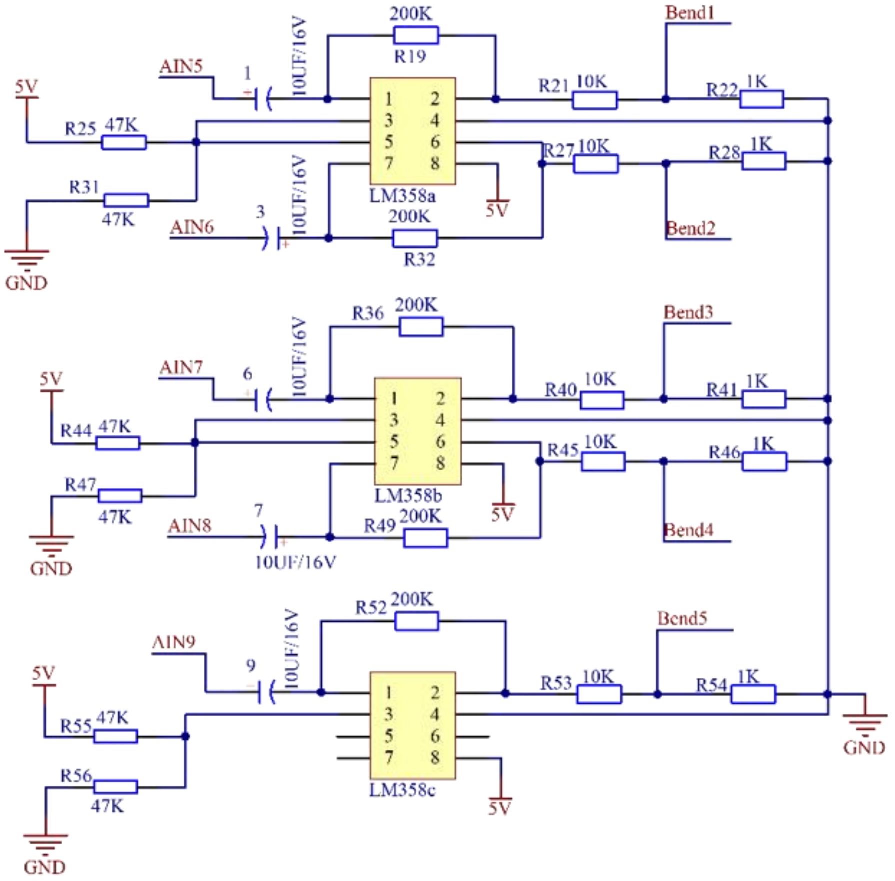


**Figure S3: The circuit schematic of the bending sensor module**

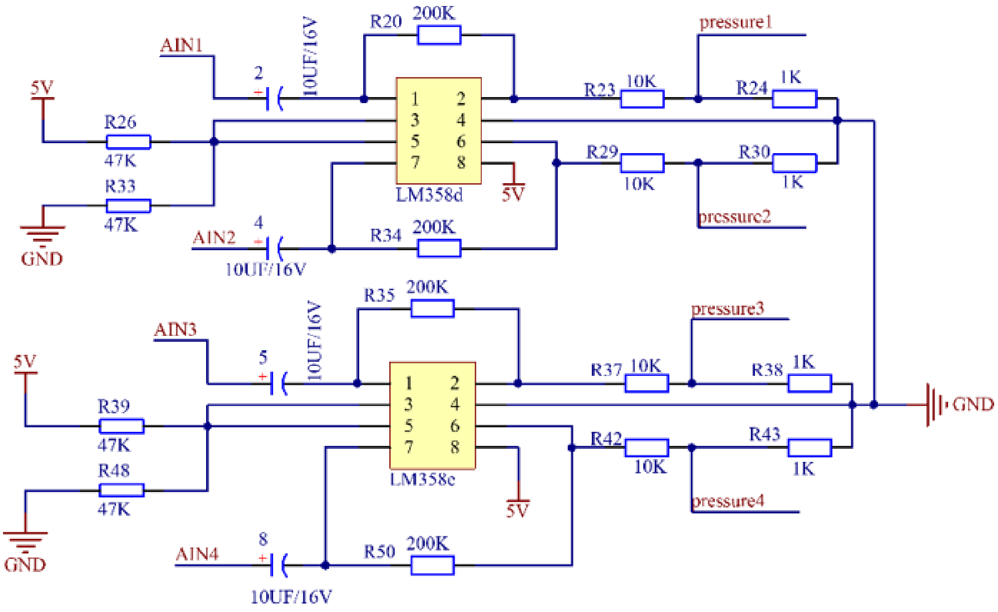


**Figure S4: The circuit schematic of the flexible thin-film pressure sensor module**

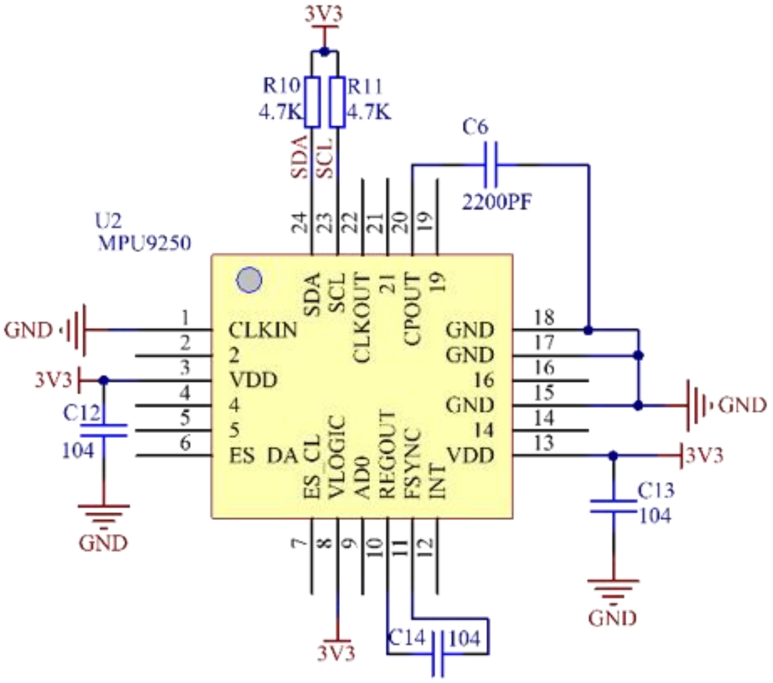


Figure S5: Pin configuration and peripheral circuit diagram of MPU-9250

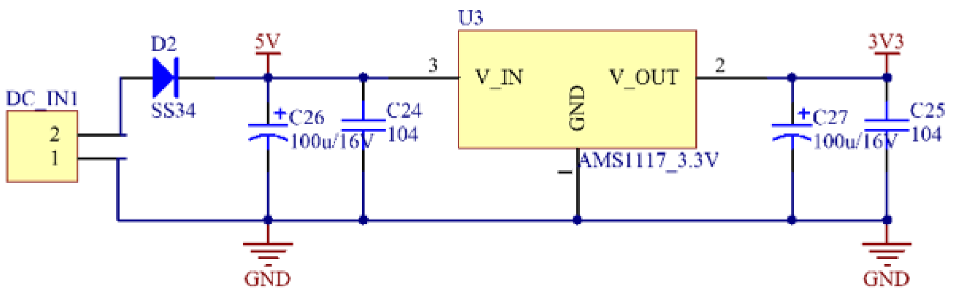


**Figure S6: The circuit schematic of the power supply module**

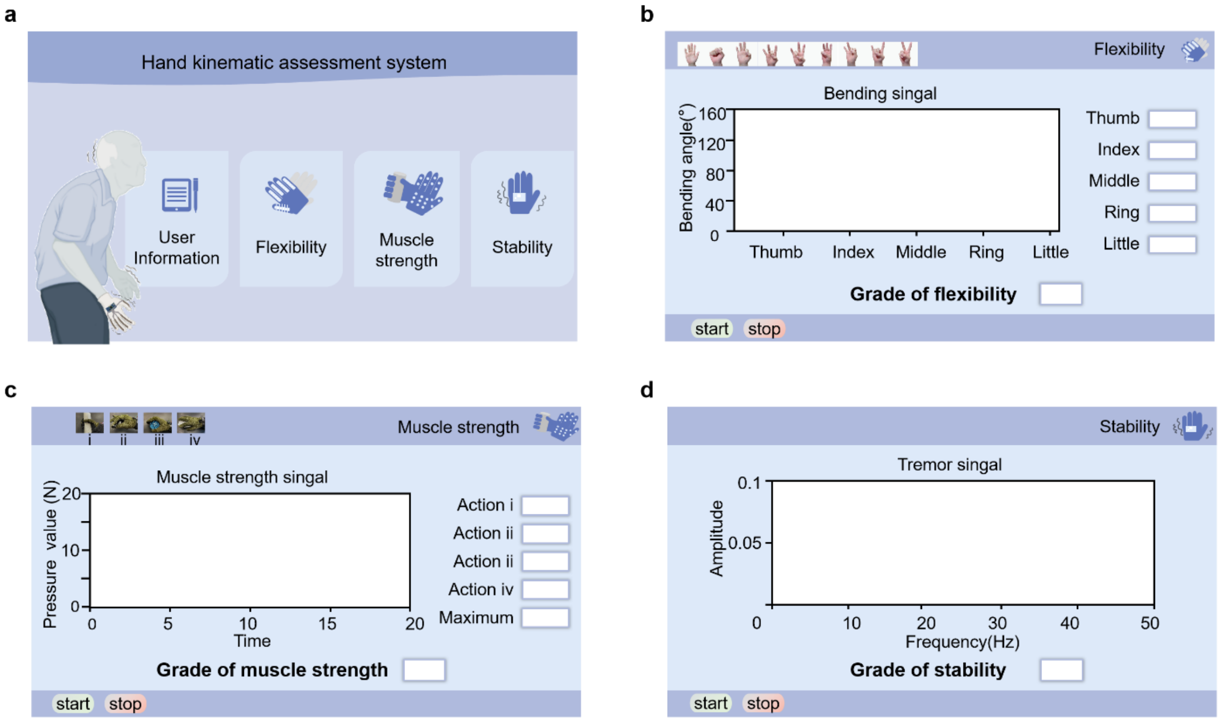


**Figure S7: Schematic diagram of the HCI interface** (a) Main interface; (b) Finger flexibility assessment interface, which is used to display the bending angle of five fingers corresponding to the completion of each gesture, the maximum bending value of five fingers, and the result of flexibility assessment; (c) Hand muscle strength assessment interface, which is used to display the completed muscle strength signal for each action, the maximum value of muscle strength, and the result of muscle strength assessment; (d) Hand stability assessment interface, which is used to display the acceleration signal of hand tremor and the stability assessment result.

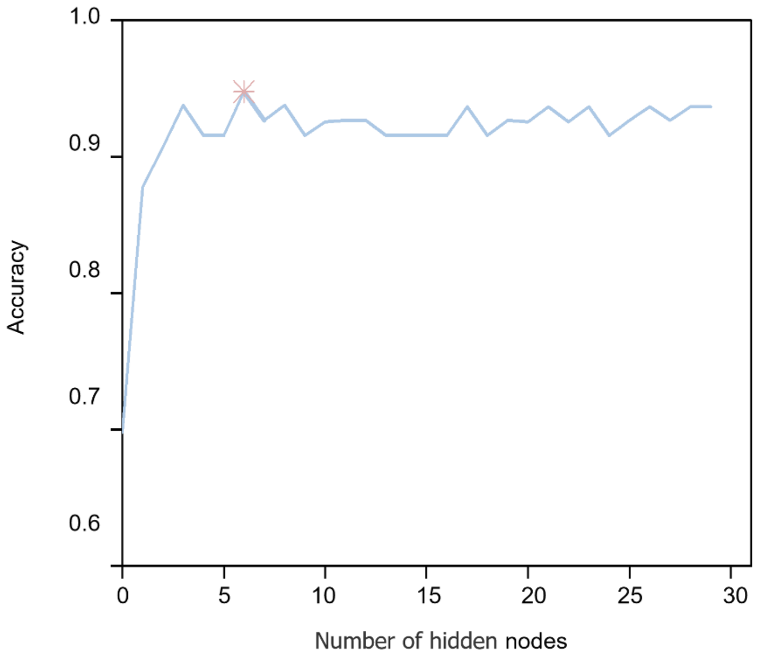


**Figure S8: Comparison of the accuracy of nodes in different hidden**

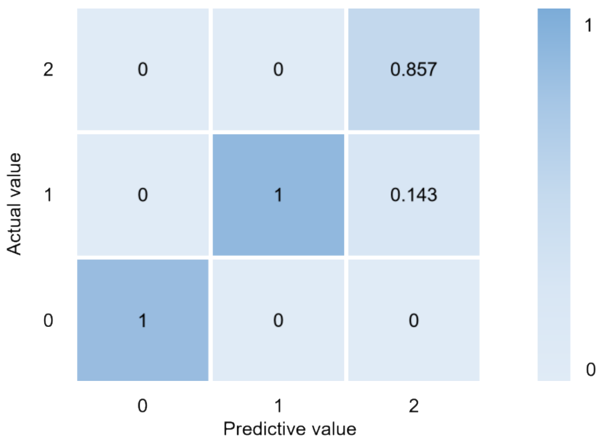


**Figure S9: Confusion matrix of tremor recognizer**

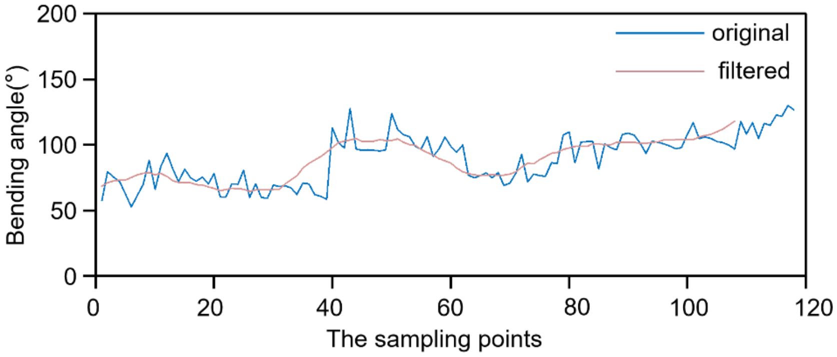


**Figure S10: Original and filtered bending signals**

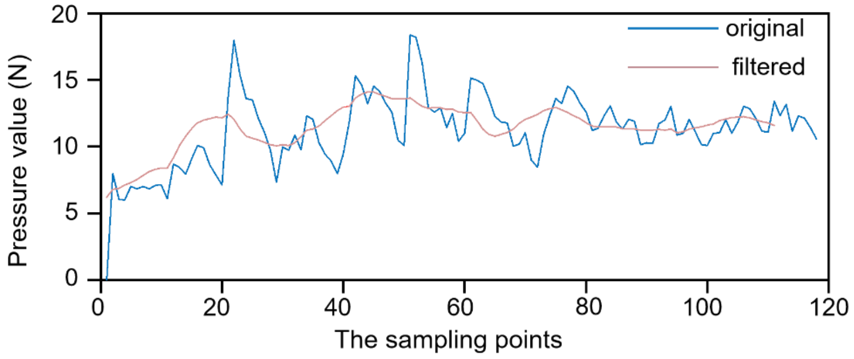


**Figure S11: Original and filtered muscle strength signals**

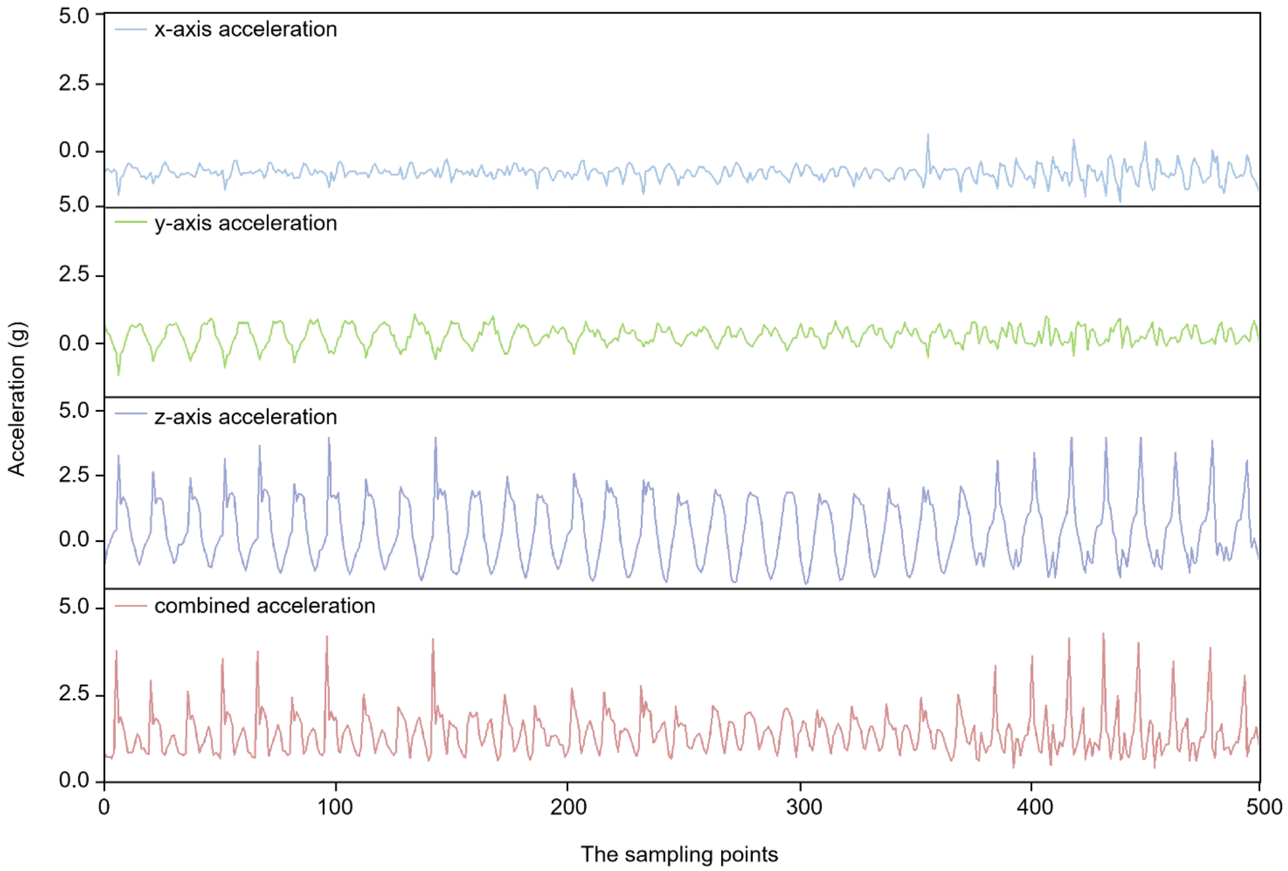


**Figure S12: Triaxial acceleration and combined acceleration of tremor signals**

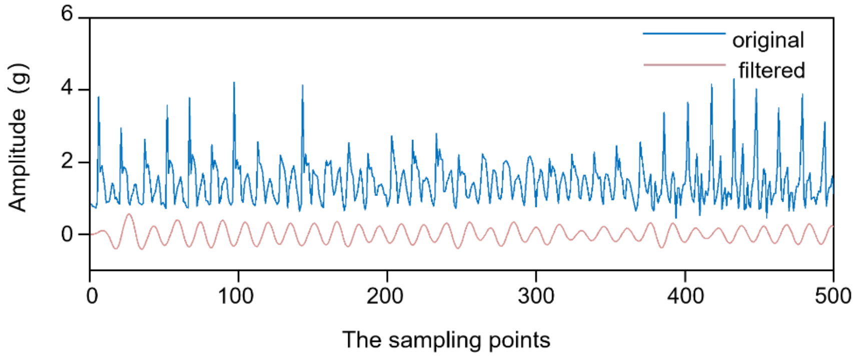


**Figure S13: Triaxial original acceleration signals and filtered tremor signals**
